# Human embryonic stem cell-derived endothelial cell product injection attenuates cardiac remodeling in myocardial infarction

**DOI:** 10.3389/fcvm.2022.953211

**Published:** 2022-10-10

**Authors:** Ana-Mishel Spiroski, Ian R. McCracken, Adrian Thomson, Marlene Magalhaes-Pinto, Mukesh K. Lalwani, Kathryn J. Newton, Eileen Miller, Cecile Bénézech, Patrick Hadoke, Mairi Brittan, Joanne C. Mountford, Abdelaziz Beqqali, Gillian A. Gray, Andrew H. Baker

**Affiliations:** ^1^Centre for Cardiovascular Science, Queen’s Medical Research Institute, University of Edinburgh, Edinburgh, United Kingdom; ^2^BHF Centre for Vascular Regeneration, University of Edinburgh, Edinburgh, United Kingdom; ^3^Edinburgh Preclinical Imaging, University of Edinburgh, Edinburgh, United Kingdom; ^4^Centre for Inflammation Research, University of Edinburgh, Edinburgh, United Kingdom; ^5^Scottish National Blood Transfusion Service, Edinburgh, United Kingdom

**Keywords:** myocardial infarction, hESC-ECP, cell therapy, scRNAseq, ligand–receptor interaction, immunomodulation

## Abstract

**Background:**

Mechanisms contributing to tissue remodeling of the infarcted heart following cell-based therapy remain elusive. While cell-based interventions have the potential to influence the cardiac healing process, there is little direct evidence of preservation of functional myocardium.

**Aim:**

The aim of the study was to investigate tissue remodeling in the infarcted heart following human embryonic stem cell-derived endothelial cell product (hESC-ECP) therapy.

**Materials and methods:**

Following coronary artery ligation (CAL) to induce cardiac ischemia, we investigated infarct size at 1 day post-injection in media-injected controls (CALM, *n* = 11), hESC-ECP-injected mice (CALC, *n* = 10), and dead hESC-ECP-injected mice (CALD, *n* = 6); echocardiography-based functional outcomes 14 days post-injection in experimental (CALM, *n* = 13; CALC, *n* = 17) and SHAM surgical mice (*n* = 4); and mature infarct size (CALM and CALC, both *n* = 6). We investigated ligand–receptor interactions (LRIs) in hESC-ECP cell populations, incorporating a publicly available C57BL/6J mouse cardiomyocyte-free scRNAseq dataset with naive, 1 day, and 3 days post-CAL hearts.

**Results:**

Human embryonic stem cell-derived endothelial cell product injection reduces the infarct area (CALM: 54.5 ± 5.0%, CALC: 21.3 ± 4.9%), and end-diastolic (CALM: 87.8 ± 8.9 uL, CALC: 63.3 ± 2.7 uL) and end-systolic ventricular volume (CALM: 56.4 ± 9.3 uL, CALC: 33.7 ± 2.6 uL). LRI analyses indicate an alternative immunomodulatory effect mediated *via* viable hESC-ECP-resident signaling.

**Conclusion:**

Delivery of the live hESC-ECP following CAL modulates the wound healing response during acute pathological remodeling, reducing infarct area, and preserving functional myocardium in this relatively acute model. Potential intrinsic myocardial cellular/hESC-ECP interactions indicate that discreet immunomodulation could provide novel therapeutic avenues to improve cardiac outcomes following myocardial infarction.

## Introduction

Heart disease is responsible for the greatest proportion of death and disability attributed to a non-communicable disease worldwide (1, 2). In addition to the staggering economic and social cost of caring for patients with ischemic heart disease in the acute setting, greater survival rates translate to increased long-term investments in recovery and rehabilitation (1, 3). The window of opportunity to halt the progression of ischemic injury is narrow, and cell-based therapies have shown potential in the treatment of ischemic heart disease, albeit largely in the pre-clinical setting (4–6). Indeed, in the pre-clinical setting, cell derivation protocols that produce a heterogeneous range of endothelial cell (EC) lineages do show some angiogenic efficacy and long-term cell retention (7). However, progenitor cell-derived products that have been reported to promote angiogenesis in small mammal models have resulted in disappointing outcomes in clinical translation to date (8). The lack of characterization of cell products and their potential effects in *in vivo* paradigms of human disease stifles effective translation in this setting. Therefore, we have focused on producing a well-characterized human embryonic stem cell-derived product, trialed in multiple pre-clinical disease paradigms with physiologically relevant, clinically translatable outcomes.

Over the last decade, we have developed a robust good manufacturing practice (GMP)-compatible human embryonic stem cell-derived endothelial cell product (hESC-ECP) that promotes angiogenesis in ischemic tissue (9–11). This robust ECP is differentiated using a well-described method and can be derived from both human embryonic and induced pluripotent stem cell (iPSC) sources. Our work with the single-source hESC-ECP is not confounded by donor variation and can be genetically manipulated, and polarization is reliable with high cell viability (10). Our recent work has focused on single-cell RNA sequencing (scRNAseq) to characterize temporal changes in transcriptional dynamics throughout derivation of pluripotent, mesodermal, mesenchymal, and endothelial lineages (11). This robust protocol produces ∼60% ECs and 40% mesenchymal cells (MESs) expressing TGFB1, FLT1, and HIF1A across both EC and MES populations, in the absence of SOX2 and POU5F1 co-expression (11). These relatively immature ECs do not specify to an arterial, lymphatic, or venous lineage and are transcriptionally distinct from fetal, infant, and adult ECs from diverse vascular beds (11). These data suggest that the resultant “unspecified” EC population, which expresses angiogenic transcription factors *in vitro*, is primed for neo-angiogenesis if introduced to an appropriate cellular milieu. Indeed, we have shown that hESC-ECP injection improves capillary endowment and perfusion as measured by using a tissue Doppler in a mouse paradigm of peripheral vascular disease, even in the presence of co-morbidities (10). In that work, we demonstrated the therapeutic potential of this cell product for acute and chronic limb ischemia, with 1 × 10^6^ cells injected intramuscularly at the time of surgery in immune-competent, -deficient, and type 2 diabetic mouse backgrounds, and injected 3 days post-surgical limb ischemia. Interestingly, superparamagnetic iron oxide nanoparticle (SPIO) magnetic resonance imaging (MRI) showed that the hESC-ECP does not persist in the hindlimb after the first week post-injection, despite gradually improving tissue perfusion compared with media-injected control mice throughout the duration of the study. PET-based 18F-FLT cell tracking suggested ∼24% of transplanted cells is retained at 4 h post-transplantation, confirmed by immunohistochemistry. By qPCR, human DNA from the hESC-ECP persists in the mouse hindlimb 1 and 7 days post-transplantation at a reduced amount, 2.5- and 4.5-fold less, respectively, but induces angiogenesis without hESC-ECP retention. Our work shows that without cellular integration of the hESC-ECP, we are able to modify the cellular milieu, shifting the tissue response toward a pro-angiogenic outcome. Progressive recovery of blood flow in the absence of long-term hESC-ECP biodistribution suggests that the angiogenic efficacy of the cell product lies in its acute effects in ischemic tissue following transplantation. This suggests that angiogenesis occurs in response to integrated cellular cues driven by the tissue microenvironment. Promoting endogenous vascular regeneration and repair in peripheral tissues alters the cellular response and subsequent functional outcomes in peripheral vascular disease (12). Therefore, we hypothesize that the hESC-ECP activates intrinsic remodeling pathways, preserving myocardial tissue and vascular endowment.

## Materials and methods

### Ethical approval

All regulated animal experiments were performed in accordance with the Animals (Scientific Procedures) Act (UK) 1986 under the auspices of the home office project and personal licenses held in the University of Edinburgh facilities, following ethical review by the University of Edinburgh Animal Welfare and Ethical Review Board (AWERB) (project 70/8933, approved 29/04/2016), approved by the Bioresearch and Veterinary Services, University of Edinburgh, and conducted in accordance with Animal Research: Reporting of *In Vivo* Experiments (ARRIVE) guidelines (13). Human ESC line H9 (WiCell, Madison, WI, USA) was used in accordance with the U.K. Stem Cell Bank Steering Committee guidelines (Project Approvals SCSC11-51 and SCSC17-26) under the guidance of Dr. Jo Mountford, Scottish National Blood Transfusion Service. All cell culture and surgical techniques are routine within the Baker Lab.

### Generation of experimental groups

To align with our established studies in HLI and chronic CAL and avoid compromising the innate response to cell injection, cardiac inflammation, and cardiotoxicity, such as with cyclophosphamide-induced immunosuppression, we used 8- to 10-week-old female Crl:CD1-Foxn1*^nu^* mice (Charles River, Edinburgh, UK). The mice were group-housed, maintained in a 12-/12-h light/dark cycle and provided free access to food and water. After 1 week of facility acclimatization, the mice were anesthetized with 50 mg/kg ketamine (Velatar, Boehringer Ingelheim, Berkshire, UK)/5 mg/kg xylazine (Rompun, 2%, Bayer, Berkshire, UK) anesthesia by intraperitoneal injection, intubated, ventilated mechanically (MiniVent 845, Harvard Apparatus Ltd., Edenbridge, UK) with positive end-expiratory pressure and 100% oxygen, and provided homeothermic support (Physitemp, Clifton, NJ, USA). Anesthetic depth was monitored with corneal and withdrawal reflexes. A left thoracotomy was performed, the pericardium opened, and a 7-0 Prolene suture (Henry Schein, Gillingham, UK) placed around the proximal left anterior descending coronary artery to induce myocardial infarction by CAL. Immediately following CAL, intracardiac injection of either hESC-ECP or un-supplemented media into the left ventricle was administered within 1 mm of the ischemic peri-infarct border. The hESC-ECP was resuspended in fresh un-supplemented EBM-2 (Lonza, Basel, Switzerland), to provide 1 × 10^6^ cells in 15 uL (CALC, *n* = 17). Either hESC-ECP (CALC) or un-supplemented EBM-2 media (CALM, *n* = 13) was injected at three sites (5 uL each) into the peri-infarct border. Warmed sterile 0.9% saline (0.2 mL) was injected into the thorax, and air removed from the chest cavity, and the thorax closed. Following skin closure with 6-0 Prolene suture (Henry Schein, Gillingham, UK), subcutaneous buprenorphine (0.05 mg/kg) was provided, anesthesia reversed with 1.0 mg/kg atipamezole (Antisedan, Henry Schein, Gillingham, UK), and an additional 0.2 mL 0.9% saline injected subcutaneously. From induction to recovery, each surgical procedure was completed in <15 min. Intubation was maintained until the mouse regained breathed spontaneously, and the blink and withdrawal reflexes were evident. Homeothermic support was provided, and following recovery, mice were group-housed in sterile individually ventilated cages with their established cage-mates, with free access to DietGel (ClearH2O, Portland, ME, USA), water, and food. The surgical sham mice (*n* = 5) underwent all procedures, except CAL and intracardiac injection. The mice were weighed and severity monitored as per facility guidance. Analgesia (0.05 mg/kg buprenorphine) and 0.5 mL warmed sterile 0.9% saline were injected subcutaneously 24 h following surgery. Intracardiac cell injection confounds using plasma cardiac troponin-I as a biomarker of infarct size. Therefore, to reduce post-surgical stress, we did not collect conscious blood samples 24 h post-surgery for troponin T analysis.

#### 14-day coronary artery ligation

**FIGURE 1:**
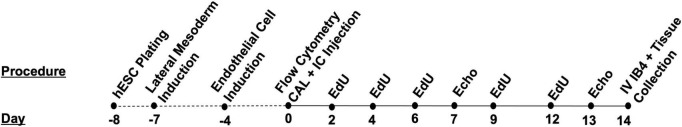
Timeline and design of human embryonic stem cell-derived endothelial cell product (hESC-ECP) differentiation and the 14-day study.

To investigate the cardiac cellular response and tissue remodeling induced by hESC-ECP injection in the ischemic mouse heart, both Crl:CD1-Foxn1nu mice were allocated to either the 14- or 1-day arm of the study. The mice allocated to the 14-day study (Figure 1) received 50 mg/kg intraperitoneal injections of Edu in 0.9% sterile saline at day 0 (0 d), 2, 4, 6, 9, and 12 d. These mice also underwent ultrasound echocardiography at d7 and d14 and received an intravenous injection of 400 ng isolectin B4 (Sigma-Aldrich, UK) in 100 uL sterile saline 15 min prior to euthanasia with 150 mg/kg intraperitoneal pentobarbital sodium (Euthatal). A subset of mice (CALM and CALC, both *n* = 6) were perfused with PBS, perfusion-fixed with 4% paraformaldehyde in PBS, and hearts were excised, weighed, and cryoprotected overnight in 30% sucrose/PBS solution at 4°C (all from Thermo Fisher Scientific, Waltham, MA, USA). The hearts were embedded in Tissue-Tek O.C.T. Compound (Sakura, Netherlands) and stored at −80°C until sectioning. Then, 10 μm sections were collected and stored at −80°C until immunohistochemistry. Tissues from the remaining mice were micro-dissected and snap-frozen in liquid nitrogen for work outside the scope of this study.

#### 1-day coronary artery ligation

The mice allocated to the 1-day arm of the study underwent a terminal procedure on 1 d post-surgery and injection. The mice (CALM, *n* = 11; CALC, *n* = 10) were anesthetized with intraperitoneal injection (50 mg/kg ketamine and 5 mg/kg, xylazine, as previously described). When the depth of anesthesia was sufficient, the aorta was catheterized, and the mouse perfused with PBS. Following PBS perfusion, Evans blue dye was perfused, and hearts were collected and processed, as previously described (14). In brief, the hearts were stained with 2% triphenyltetrazolium chloride (TTC) to assess the cardiac risk area and infarct area. The stained hearts were cut into 1-mm sections transverse to the apex/base axis to the level above the suture and incubated in 2% TTC for 30 min at 37°C. The hearts were blotted dry and post-fixed in 4% formalin. The sections were scanned, and risk and the infarct area were quantified manually in Fiji. The CALD mouse that survived 1 d post-surgery underwent this procedure as well.

### Human embryonic stem cell-derived endothelial cell product differentiation

H9 (P43-47) hESCs were differentiated to an hESC-ECP, as previously described (10, 11). In brief, hESCs maintained in hESC StemPro SFM media (Thermo Fisher Scientific, Waltham, MA, USA) on a vitronectin matrix (Life Technologies, Paisley, UK) were dissociated at day 0 and seeded onto a fibronectin matrix (Sigma, St. Louis, MO, USA) in mTeSR1 media (STEMCELL Technologies, BC, Vancouver, Canada) supplemented with 10 μM ROCK inhibitor (Y27632) (Tocris, Bristol, UK). The lateral mesoderm was induced at day 1 with 25 ng/ml BMP4 (R&D Systems, Minneapolis, MN USA) and 7 μM GSK3 inhibitor CHIR99021 (Sigma, St. Louis, MO, USA) in N2B27/neurobasal/DMEM:F12 media (Life Technologies, Paisley, UK). At day 4, endothelial fate was induced with 200 ng/ml VEGF (R&D Systems, Minneapolis, MN, USA) and 2 μM Forskolin (Sigma, St. Louis, MO, USA) in StemPro34 media (Life Technologies, Paisley, UK), with a fresh media change at day 5. At day 6, cells were plated into a matrix-free T75 flask and maintained with daily media changes through day 8 with EGM-2 media (Lonza, Basel, Switzerland) supplemented with VEGF (50 ng/ml) and 1% human AB serum (Sigma, St. Louis, MO, USA). At day 8, the cells were visualized with the EVOS XL Core Cell Imaging System (Thermo Fisher Scientific, Waltham, MA, USA), detached with 1X TrypLE Express Enzyme (Thermo Fisher Scientific, Waltham, MA, USA), and kept on ice for flow cytometry assessment and *in vivo* use.

A group of mice (*n* = 6) were randomly allocated to receive freeze-thaw-killed (6) hESC-ECP injection (CALD). Only one mouse survived the procedure. This aspect of the study was discontinued to ensure alignment to ethical guidelines.

### Cell staining and flow cytometry

At day 8, the cells were mixed 1:1 with trypan blue to identify dead cells, visualized, quantified (Bio-Rad, Hertfordshire, UK), and characterized for pluripotency and endothelial cell phenotype, as previously described (11). In brief, the cells were stained for pluripotency (SSEA-3 +/TRA-160 +) with PE rat anti-human SSEA-3, PE Rat IgM, κ isotype control, Alexa Fluor^®^ 647 Mouse anti-Human TRA-1-60, Alexa Fluor^®^ 647 Mouse IgM, and endothelial markers (CD31 +/CD144 +) with PE mouse anti-human CD144, PE mouse IgG1 κ isotype control (BD Biosciences), and APC anti-human CD31 and APC mouse IgG1 κ isotype control (both eBioscience). Flow was conducted with an Attune NxT system (Thermo Fisher Scientific, Waltham, MA, USA) and data analyzed using FlowJo software (FlowJo LLC, Ashland, OR, USA). Preparations with an acceptable EC population (>60% EC, remaining cells mesenchymal) were used for this study.

### Echocardiography

At day 8, the cells were mixed 1:1 with trypan blue to identify dead cells, visualized, quantified (Bio-Rad, HertfordshiCardiac function was assessed by ultrasound echocardiography with Doppler flow under isoflurane anesthesia (4% induction, ∼1.75% maintenance) on the Vevo 3100 Preclinical Imaging System and analyzed in Vevo Lab V3.2.6 image analysis software (FUJIFILM VisualSonics, Inc., Toronto, Canada). Post-CAL cardiac function (d7) was assessed to determine early post-injection cardiac function and late function at d14. Left ventricle (LV) function was assessed with brightness mode (B-mode), pulse wave Doppler (PWD), and motion mode (M-mode) in parasternal long axes, short axes and apical 4 chamber view (as appropriate), EKV (ECG-gated kilohertz visualization), and global longitudinal strain (GLS) in the long axis. All echocardiography analyses were blinded.

### Immunohistochemistry

Slides were placed in ice-cold acetone for 10 min, followed by Masson’s trichrome (MT) staining, as per the manufacturer’s instructions (Thermo Fisher Scientific, UK) to investigate the infarct area. Collagen was stained using picrosirius red (PSR) for 1 h. Images were acquired in brightfield for MT and PSR on an Axioscan 2.1 slide scanner (Zeiss, Cambridge, UK), tiled at 40x, and the infarct and collagen areas (%) quantified manually in Fiji.

#### Immunofluorescence

Tissue staining was performed in blocking solution [0.5% Triton-X-100, 10% normal goat serum (NGS, Thermo Fisher Scientific)], and 1% bovine serum albumin. For antibodies raised in mouse, Mouse-on-Mouse blocking reagent (Vectorlab, MKB-2213-1) was used, as per the manufacturer’s protocol. A range of conjugated and unconjugated antibodies were used (Table 1). In brief, the sections were stained overnight at 4°C or for 2 h at room temperature, then washed, and incubated with corresponding secondary antibodies for 2 h at room temperature. The sections were mounted in Fluoromount-GTM with DAPI (Invitrogen). Fluorescence microscopy images were obtained using a slide scanner Axioscan 2.1 (Zeiss, Cambridge, UK). Vessel density was quantified as the percentage of Isolectin IB4^+^ cells measured at the infarct border using a vessel density Fiji macro. For Edu quantification, after Click-iT Edu Imaging Kit with Alexa Fluor 647, Edu^+^/Troponin T^+^ cells were manually counted at the infarct border.

**TABLE 1:** Immunofluorescence antibodies and dilutions.

Primary antibodies (dilution)	Manufacturer
Isolectin GS-IB4 (*Griffonia simplicifolia)* α-biotin Strep Conjugate	Thermofisher, 121414
Troponin T (1:40)	Invitrogen, MA5-12960
α-Smooth Muscle Actin Cy3 (1:100)	Sigma, C6198

**Secondary antibodies (dilution)**	**Manufacturer**

Streptavidin, Alexa Fluor^Tm^ 647 conjugate	Thermo Fisher, S21374
Goat anti mouse 568 (1:300)	Invitrogen, A-11004

### Ligand–receptor interaction analysis

To investigate the potential hESC-EC and -MES ligand–receptor interactions (LRIs) from our previously published hESC-ECP dataset (GEO; GSE131736), we mined a publicly available (ArrayExpress: E-MTAB-7895) 10x Chromium C57BL/6J mouse cardiomyocyte-free scRNAseq dataset from naive, 1-, and 3-day CAL mouse hearts (15) with CellPhoneDB (16), similar to network analysis conducted by Wang and colleagues in ligated neonatal hearts (17).

CellPhoneDB (16) was used to identify potential ligand–receptor interactions (LRIs) between the hESC-ECP (GEO; GSE131736) and resident cardiac populations in a publicly available C57BL/6J mouse cardiomyocyte-free scRNA-seq dataset from naive, 1-, and 3-day CAL mouse hearts (15).

Data for the mouse cardiac MI were downloaded from ArrayExpress (E-MTAB-7895) and subsequently processed using the 10X Cell Ranger pipeline (v3.1.0) with the 10X pre-built Cell Ranger mm10-3.0.0 reference. Custom quality control was performed using the Scater package (18) using data from the standard filtered matrices. Cells with a total UMI count exceeding 4 median absolute deviations (MADs) from the median value or with fewer than 200 genes identified were removed from downstream analysis. In addition, the cells with a high proportion of counts from mitochondrial genes (>4 MADs) were also excluded. Prior to merging datasets, data normalization was performed using the MultiBatchNormalisation (19) to minimize batch effects between datasets. The top 2,000 most variable genes were subsequently identified using the standard FindVariableFeatures method (20). Normalized data were then scaled using the standard ScaleData method (20) and principal component analysis conducted using the previously identified variable genes. Harmony correction was implemented when integrating datasets, following the standard workflow (21). The cells were clustered and projected using UMAP implementing the standard Seurat workflow and utilizing the corrected harmony reduction embedding values (20). Clusters were annotated based on the cluster marker genes, as described previously (15). Day 8 hESC-ECP scRNA-seq data were processed, as previously described (11).

At day 8, the cells were mixed 1:1 with trypan blue to identify dead cells, visualized, quantified (Bio-Rad, HertfordshiHuman orthologs for mouse genes were identified using the BioMart package (22) and used to replace mouse gene names in the dataset in order to run CellPhoneDB ligand–receptor analysis (16). Mouse genes with no identified human ortholog were removed from downstream analysis. Count values from the mouse cardiac MI data were then merged with count values from the hESC-ECP data, and data normalization performed using the standard Seurat NormalizeData method (20). Normalized count data were then used as input into the CellPhoneDB package, which was run using the statistical method with 100 iterations and a *P*-value threshold of 0.05.

### Statistical analyses

At day 8, the cells were mixed 1:1 with trypan blue to identify dead cells, visualized, quantified (Bio-Rad, HertfordshiPower calculations were performed to determine the minimum sample size required to achieve statistical significance for left ventricular (LV) function at 14 d. For CAL procedures, with a power of 80% and a 5% chance of type I error, 12 mice/group are required. At an 80% survival rate, 15 mice/group are needed.

Data were analyzed in JMP 12 (SAS Institute, Inc., Cary, North Carolina, USA). Distribution was verified with the Shapiro–Wilk test, and non-parametric data were log-transformed, where necessary. Echocardiography outcomes were analyzed by ANOVA with Tukey’s *post-hoc* testing, where appropriate. Student’s *t*-test was conducted, where appropriate. Data were graphed in GraphPad Prism 9 (GraphPad Software Inc., USA) and are presented as the mean ± SEM and significance indicated.

## Results

### Human embryonic stem cell-derived endothelial cell product differentiation is efficient and reproducible

**FIGURE 2:**
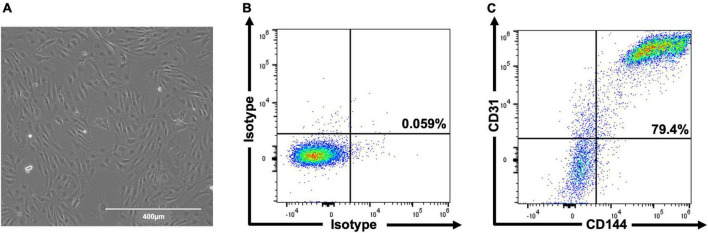
Representative images of hESC-ECP differentiation and phenotypic characterization. Day 8 morphology and confluency **(A)** and representative flow cytometric analysis **(B,C)** of isotype controls and hESC-ECP stained for CD31 (PECAM1) and CD144 (VE-cadherin).

In total, three differentiations were produced. Cells (Figure 2A) comprised 69.6% ± 4.0 CD31^+^/CD144^+^ (EC) at day 6 and 83.5% ± 3.3 at day 8 (Figures 2B,C). Staining for pluripotent markers at day 8 revealed cells to be 0.08% ± 0.01 TRA160^+^/SSEA3^+^ (Supplementary Figure 1). Finally, 2.6 × 10^6^ cells were harvested per T75 flask with 97% ± 0.6 viability.

### Human embryonic stem cell-derived endothelial cell product injection reduces risk area, but not initial infarct area

**FIGURE 3:**
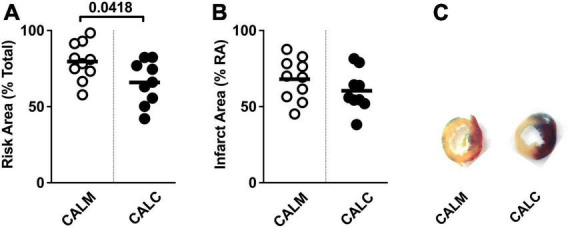
1-Day infarct area following hESC-ECP or media injection. Risk area (%) of LV area below the suture **(A)**, infarct area,% **(B)** within the risk area, and **(C)** representative sections 1 d post-infarct in CALM, *n* = 11, and CALC, *n* = 10 groups. Student’s *t*-test; data are presented as individual points and as mean ± SEM.

At 1 day post-injection, the infarct risk area was significantly reduced in the CALC mice compared with CALM (Figure 3A), while there was no difference in the infarct area at 1 day post-ligation in CALM and CALC mice (Figure 3B), as determined by TTC staining (Figure 3C). The infarct area of the CALD mouse that survived to 1 d post-injection was 88% of the risk area and 75% of the total left ventricle area.

### Human embryonic stem cell-derived endothelial cell product injection preserves left ventricular function and structure

**FIGURE 4:**
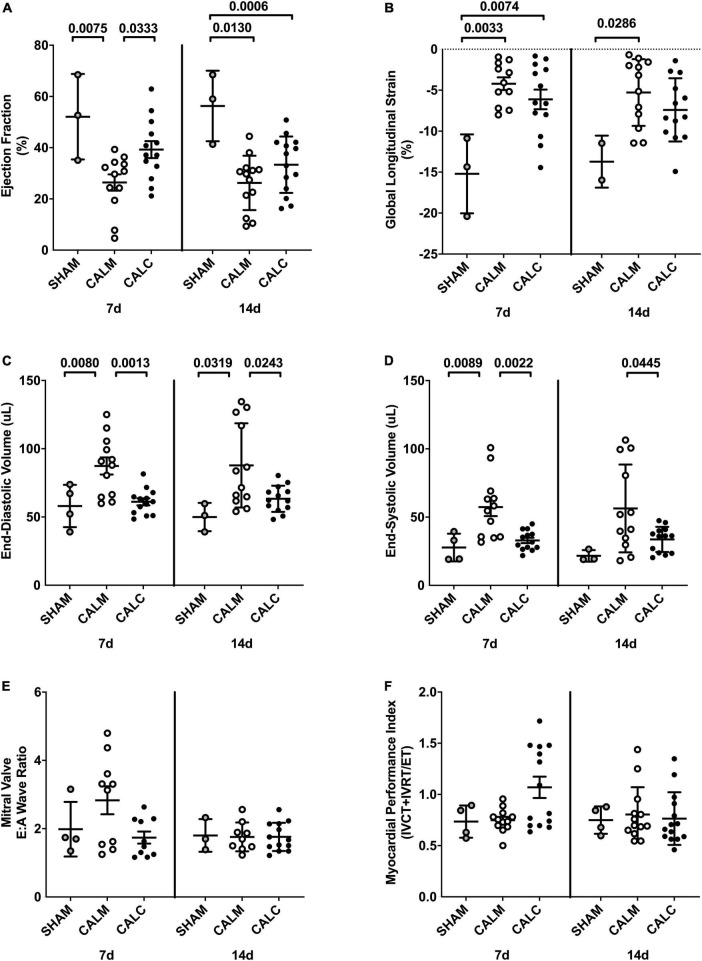
Echocardiography outcomes in hESC-ECP and media injected mice. Ejection fraction **(A)** was preserved in CALC mice compared to CALM at 7 d, but not at 14 d post-injection. Global longitudinal strain **(B)** was increased at 7 d in both CALC and CALM compared with SHAM, but only in CALM mice at 14 d. End-diastolic volume, EDV **(C)**, and end-systolic volume, ESV **(D)**, were preserved at 7 and 14 d in CALC compared with CALM mice. Mitral valve E/A wave ratio **(E)** and myocardial performance index **(F)** were not different amongst groups at either time point. ANOVA with Tukey’s *post-hoc* test. Data are mean ± SEM.

Cell injection prevented CAL-induced cardiac dilation at 7 and 14 days post-injection (Figures 4C,D), preserved ejection fraction at 7 days, and slowed progression of reduced ejection fraction in CALM mice at 14 days (Figure 4A). While there were no observed differences in the mitral valve E/A wave ratio (Figure 4E) or myocardial performance index (Figure 4F) amongst groups, global longitudinal strain analysis suggested that hESC-ECP injection slowed the progression toward heart failure (Figure 4B).

### Human embryonic stem cell-derived endothelial cell product injection reduces infarct area and collagen deposition at 14 days

**FIGURE 5:**
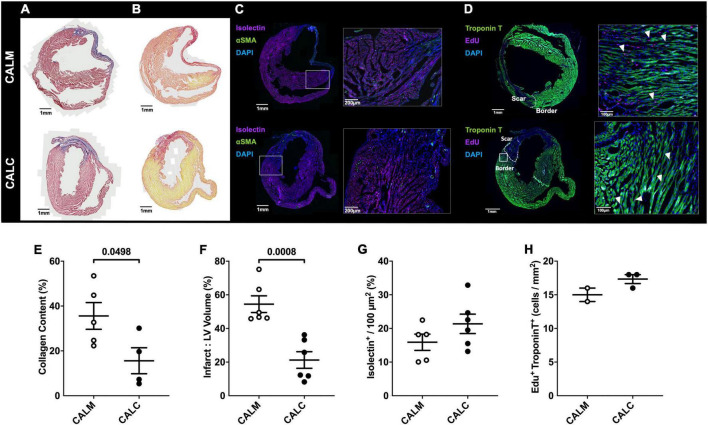
Immunohistochemical assessment of mature infarct area and myocardial characteristics. Collagen deposition **(A,E)** and infarct volume **(B,F)** were significantly reduced in CALC mice compared with CALM. There were no differences in isolectin B4 perfused vascular density **(C,G)** or myocardial regeneration **(D,H)** between the groups. Student’s *t*-test; data are mean ± SEM. White arrows **(D)** indicate Edu + Troponin T + cells.

In order to study the effect of hESC-ECP injection on cardiac remodeling, we performed immunohistochemical assessment of the mature infarct area at 14 days by MT and PSR staining. Cell injection reduced collagen deposition (CALM: 35.6 ± 6.0%, CALC: 15.6 ± 5.8%) (Figures 5A,E), and infarct volume was significantly reduced in the CALC mice compared with CALM (CALM: 54.5 ± 5.0%, CALC: 21.3 ± 4.9%) (Figures 5B,F). There were no differences in isolectin B4 perfused vascular density (Figures 5C,G) or myocardial regeneration (Figures 5D,H) between the groups.

### Ligand–receptor interaction analysis reveals predicted interactions between the human embryonic stem cell-derived endothelial cell product and resident post-coronary artery ligation cardiac populations

**FIGURE 6:**
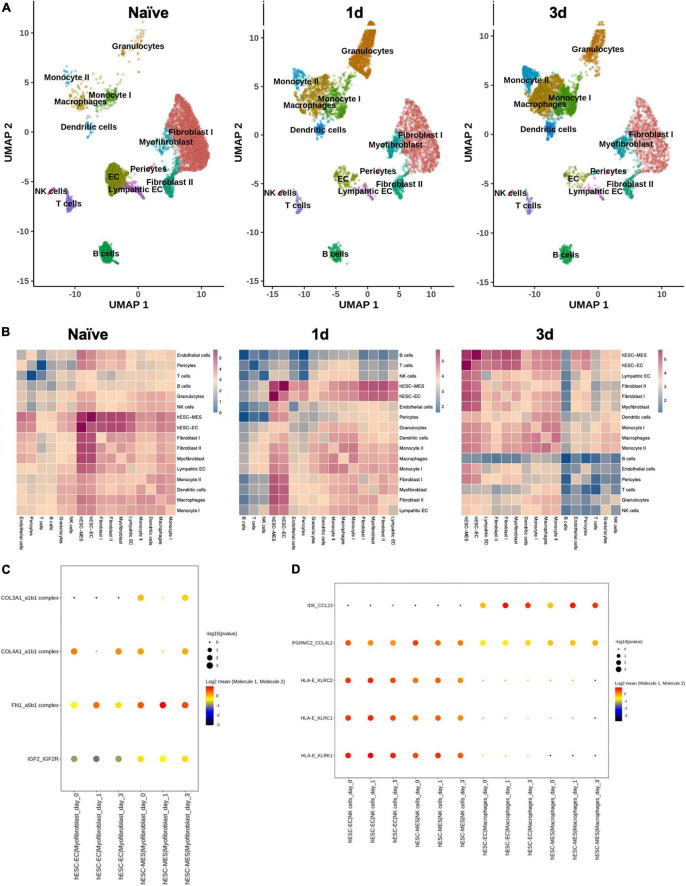
Ligand–receptor interactions between hESC-ECP constituents and resident cardiac populations. **(A)** UMAP projection of clusters identified from cardiomyocyte-free scRNA-seq data of C57BL/6J hearts taken either prior to CAL (naive), or 1 or 3 days post-CAL. **(B)** Ligand–receptor interaction (LRI) analysis between day eight hESC-ECP populations and resident cardiac populations at each timepoint. Heatmaps illustrate log_10_(number of predicted LRI) between each population. Dot plots of LRIs between hESC-ECP populations and **(C)** myofibroblasts and **(D)** NK and macrophages.

Dimension reduction and clustering analysis of cardiomyocyte-free scRNA-seq data from C57BL/6J naive, 1 day, and 3 days post-infarcted hearts identified populations comparable to those originally identified by Forte et al. (15) (Figure 6A). Following merging with data from the day 8 hESC-ECP (11), ligand–receptor interaction (LRI) analysis predicted multiple interactions between component populations of the day 8 hESC-ECP (EC and mesenchymal) and resident cardiac immune (NK and macrophage) and stromal (myofibroblast and fibroblast) populations (Figure 6B). In keeping with our observation of modified infarct formation, both EC and mesenchymal populations of the hESC-ECP expressed fibronectin (*FN1*) and collagens (*COL3A1* and *COL4A1)* known to form complexes with integrins to modulate myofibroblast function (23) (Figure 6C). Both hESC-ECP component populations also expressed the *HLA-E* ligand, known to supress NK activity *via* the KLRD1/KLRC1 receptor (24).

## Discussion

This study shows that hESC-ECP injection in the infarcted heart improves functional and structural outcomes in a mammalian paradigm of heart failure. LR complexes within immune and myo-/fibroblast niches suggest a potential mechanism for pathological reprogramming with delivery of live hESC-ECP independent of cellular integration. Potentially due to time-dependent LRIs between live hESC-ECs and -MESs, and resident endothelial cells, monocytes, macrophages, fibroblasts, and myofibroblasts, the acute tissue remodeling window through 3 days post-ligation is a powerful and dynamic environment in which to effect change in the infarcted heart.

Through ultrasound echocardiography and longitudinal strain analyses, we demonstrate that intracardiac hESC-ECP injection (CALC) of 1 × 10^6^ cells within the peri-infarct region in the CAL model of myocardial infarction (MI) preserves the left ventricle ejection fraction (EF) and reduces ventricular dilation 14 days post-injection compared with media-injected (CALM) in Crl:CD1-*Foxn1^nu^* mice. Greater end-diastolic volume (EDV) and end-systolic volume (ESV) in the CALM mice suggest that hESC-ECP injection preserves functional myocardium and abrogates CAL-induced ventricular dilation. Additionally, we demonstrate that at 1 day post-surgery, the infarct area is comparable in the CALC and CALM mice, suggesting that subsequent scar remodeling and myocardial function are independent of initial infarct size. Analyses suggest that hESC-ECP injection may influence acute myocardial survival, fibroblast maturation, and preserve contractile myocardium 14 days post-treatment, which integrates into the mature scar. These first studies in the setting of MI suggest that improved functional outcomes in parallel with clear, time-bound histological changes are indicative of divergent tissue remodeling pathways in hESC-ECP-injected mice.

Recent pre-clinical testing of other stem cell-based therapies suggests a complex mechanism of efficacy (5, 25). Rather than providing a consistent, well-described outcome in the preservation or recovery of cardiac tissue, these interventions demonstrate diverse effects on resident cardiac cell populations, inducing localized immune activation (15, 26), potentially *via* paracrine mechanisms (27). Vagnozzi and colleagues have recently reported that transplanted mouse-on-mouse cell products enhance inflammation and direct immune cell trafficking, modulating the chronic inflammatory profile post-MI due to cell death (6). However, based on our preliminary LRI, this conclusion may not be fully pertinent herein and not be generalizable to all stem cell-based therapies. The potential for direct interaction with resident cell populations suggests that a viable cell product may be necessary. LRI analyses suggest that the hESC-EC and -MES have the potential for cell–cell crosstalk with stromal and immune cell types, including cell signaling, that may influence immune cell recruitment and infiltration into the injured heart (CCL23, CCL4L2, KLRC1, KLRK1) and cardiac remodeling (IGF2R and collagen– and fibronectin–integrin complexes a1b1 and a5b1, respectively). Although historically transgenic immunocompromised mice have been used in cardiac cell therapy studies to avoid immune-induced xenograft failure and the effects of pharmacological immunosuppression, recent work suggests that transgenic mice are able to mount a robust immune response in CAL (28). Characterization of the modified LRI response in transgenic immunocompromised mice would help identify the biological factors influencing disease severity in CAL (15).

The extent of injury, quality of repair, and the breadth of myocardial remodeling are intricately linked to the intensity of the inflammatory response. Ischemic injury leads to the dynamic recruitment and mobilization of a range of innate and adaptive immune cells that contribute to the development of a mature scar, and modification of fibroblast and myofibroblast remodeling. As fibroblast proliferation and immune cell activation peak 2–4 days post-MI (15, 29), future investigations of the early post-treatment therapeutic response could identify druggable networks during the acute remodeling window. Neutrophils are initially recruited to clear necrotic tissue, in our studies peaking in the heart 24–48 h post-MI. Ly6C^high^ monocytes, precursors of the “classical” inflammatory macrophage, are recruited from splenic and bone marrow reservoirs and localize to the area of damage. Macrophages dominate during the early immune response, clearing apoptotic neutrophils and necrotic debris by 3–7 days post-infarct. Accumulating monocytes give rise to tissue macrophages with a reparative phenotype, initiating neo-angiogenesis and fibroblast collagen production, while attenuating inflammation. Within several weeks, monocyte recruitment subsides and a mature scar forms within the infarcted region (26, 30, 31). The dynamic post-MI inflammatory response is influenced by immune cell localization and expansion of immune cells within the associated pericardial adipose tissue, an organ rich in B cells (32–36). Adverse remodeling can be caused by excessive or deficient inflammatory cell recruitment into the heart post-MI (26, 30, 31, 37). Thus, targeted immunomodulation offers a powerful approach to improve pathological cardiac remodeling. hESC-ECP therapy could serve to reprogramme subsequent scar formation *via* inflammatory or immune mechanisms, modifying fibroblast remodeling and preserving functional myocardium, subsequently improving clinically relevant physiological outcomes.

It is important to note the limitations of this study. We have focused on the effect in a relatively acute setting; thus, extended long-term studies need to be performed in the future to elucidate downstream effects in a more translational paradigm of heart failure. While we provide evidence of potential mechanisms of action, we have not formally assessed this and confirmed *via* independent techniques. Additionally, our ECP is a combination of both endothelial-like and mesenchymal-like cells (10, 11), and we do not know which component, or indeed if both populations, are required for the phenotype. Our utilization of dead cell materials, which has previously been suggested to induce immune-mediated remodeling, was unsuccessful and would require extensive development in the future. Furthermore, we were not able to demonstrate cellular integration. However, in a prior study, in the setting of peripheral limb ischemia, we demonstrated that our ECP is not likely to survive a prolonged period of time (beyond 24 h; 75% lost at 24 h) (10); thus, assessing cellular integration is unlikely to provide evidence of causality. Further studies will address these issues.

## Data availability statement

The original contributions presented in this study are included in the article/Supplementary material, further inquiries can be directed to the corresponding author.

## Ethics statement

The animal study was reviewed and approved by the University of Edinburgh Animal Welfare and Ethical Review Board (AWERB), under project (70/8933, approved 29/04/2016).

## Author contributions

A-MS, IM, MB, JM, GG, and AHB: conceptualization. A-MS, IM, MM-P, AT, MB, JM, GG, and AHB: methodology. A-MS, IM, and MM-P: formal analysis. A-MS, MM-P, and AT: investigation. A-MS, GG, and AHB: resources. A-MS and IM: data curation and visualization. A-MS, MM-P, and CB: writing – original draft preparation. A-MS, JM, PH, GG, and AHB: supervision. A-MS and GG: project administration. MB and AHB: funding acquisition. All authors contributed to writing – review and editing, read, and agreed to the published version of the manuscript.

## Supplementary material

Supplementary Figure 1 Day 8 representative flow cytometric analysis of **(A)** isotype controls and **(B)** hESC-ECP stained for pluripotent markers TRA160C and SSEA3C.

## Abbreviations

ARRIVE: Animal Research: Reporting of *In Vivo* Experiments
AWERB: Animal Welfare and Ethical Review Board
CAL: coronary artery ligation
CCL23: C-C motif chemokine ligand 23
CCL4L2: C-C motif chemokine ligand 4 like 2
EC: endothelial cell
EdU: 5-ethynyl-2 ′ -deoxyuridine
EDV: end-diastolic volume
EF: ejection fraction
EKV: ECG-gated kilohertz visualization
ESV: end-systolic volume
FLT1, Fms-related receptor tyrosine kinase 1: vascular endothelial growth factor receptor 1
GLS: global longitudinal strain
GMP: good manufacturing practice
hESC-ECP: human embryonic stem cell-derived endothelial cell product
HIF1A: hypoxia inducible factor 1 subunit alpha
IGF2R: insulin-like growth factor 2 receptor
iPSC: induced pluripotent stem cell
KLRC1: killer cell lectin-like receptor C1
KLRK1: killer cell lectin-like receptor K1
LRI: ligand–receptor interaction
LV: left ventricle
Ly6C: lymphocyte antigen 6C
MES: mesenchymal cells
MI: myocardial infarction
MT: Masson’s trichrome
NK: natural killer cells
PBS: phosphate-buffered saline
PFA: paraformaldehyde
PSC: pluripotent stem cell
POU5F1: POU class 5 homeobox 1
PWD: pulse-wave Doppler
scRNAseq: single-cell RNA sequencing
SOX2: SRY-box transcription factor 2
SPIO: superparamagnetic iron oxide nanoparticle
SSEA-4: stage-specific embryonic antigen 4
MRI: magnetic resonance imaging
PSR: picrosirius red
TGFB1: transforming growth factor beta 1
TRA-181: podocalyxin
TTC: triphenyltetrazolium chloride
VEGF: vascular endothelial growth factor.
