# Supplementary figures and images for: Human embryonic stem cell-derived endothelial cell product injection attenuates cardiac remodeling in myocardial infarction
Source: Front Cardiovasc Med. 2022 Oct 10;9:953211. doi: 10.3389/fcvm.2022.953211 (PMC9588936; doi:10.3389/fcvm.2022.953211)

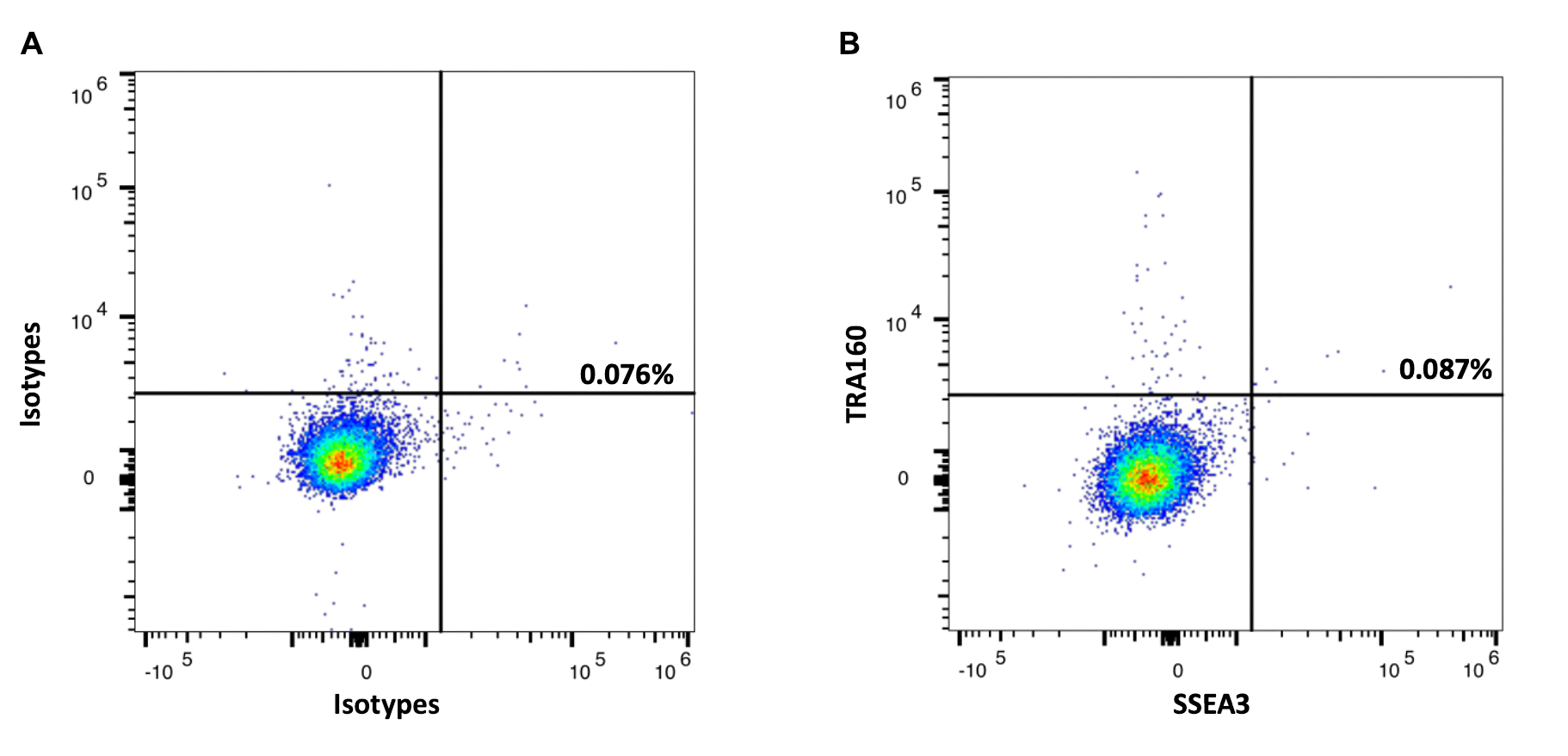

Supplement: Supplementary Figure 1 — Day 8 representative flow cytometric analysis of (A) isotype controls and (B) hESC-ECP stained for pluripotent markers TRA160C and SSEA3C. [file Image_1.TIF]
